# Marginal Discrepancy of Five Contemporary Dental Ceramics for Anterior Restorations

**DOI:** 10.1055/s-0042-1758787

**Published:** 2023-01-04

**Authors:** Noor Nawafleh, Muhanad Hatamleh, Yasmeen Janzeer, Ali Alrahlah, Khold Alahadal

**Affiliations:** 1 Department of Applied Dental Sciences, Faculty of Applied Medical Sciences, Jordan University of Science and Technology, Irbid, Jordan; 2 Department of Applied Medical Sciences, Luminus Technical University College, Amman, Jordan; 3 Engineer Abdullah Bugshan Research Chair for Dental and Oral Rehabilitation, College of Dentistry, King Saud University, Riyadh, Saudi Arabia; 4 Restorative Dental Sciences Department, College of Dentistry, King Saud University, Riyadh, Saudi Arabia

**Keywords:** marginal fit, marginal accuracy, CAD/CAM, zirconia, lithium silicate

## Abstract

**Objectives** This study aimed to compare marginal accuracy of five contemporary all-ceramic crowns indicated for anterior restorations.

**Materials and Methods** A master die of maxillary central incisor was prepared for all-ceramic crown and duplicated to produce 50 replicas of epoxy resin material. Five ceramic materials were used to mill the crowns ( *n* = 10). All crowns were manufactured following the same digital workflow; same master die, scanning unit and design software, and the recommended manufacturing protocol. Final seating of crown was secured by a small droplet of temporary cement on its incisal edge. Marginal accuracy was evaluated by scanning electronic microscope with a magnification of 300 × . Vertical marginal gap was measured for each crown at predefined four points.

**Statistical Analysis** One-way analysis of variance was used to test differences between groups and Tukey test was used for multiple comparisons between group combinations. A level of significance at 95% was set for all statistics.

**Results** The highest mean marginal gap and mean maximum gap calculated were for the e.max CAD crowns (49.2 µm, 87.6 µm), while the lowest values were for the Cercon xt crowns (10.2 µm, 21.7 µm). The mean marginal gap and the mean maximum gap of the e.max CAD crowns were statistically significantly greater than those of all other groups ( *p* < 0.05). However, the differences between all other combinations were insignificant ( *p* > 0.05).

**Conclusion** Marginal accuracy of lithium disilicate crowns is clinically acceptable. Zirconia and zirconia-reinforced lithium silicate materials can produce a greater level of marginal accuracy compared to lithium disilicate.

## Introduction

Marginal accuracy of fixed prosthodontics is heavily researched as it determines their clinical success. 1 2 3 4 5 6 7 8 A maximum cement film thickness of 25 to 40 µm was identified as set by the American Dental Association (ADA). 9 In spite of the absence of a clear evidence that a certain method of fabrication provides a consistently superior marginal fit, 10 a gap of 25 to 40 µm is hard to achieve with the conventional fabrication processes because of the various materials and clinical and laboratory procedures involved. However, the increased popularity of computer-aided design and computer-aided manufacturing (CAD/CAM) technologies and the development of novel microstructures of ceramic materials have improved the fixed prosthodontics practice including the achievable marginal gap. 11 12 13

Aesthetics has increasingly become a great influence in choosing restorative materials even in the posterior region. Lithium disilicate (LD) might be considered the most attractive monolithic all-ceramic alternative for anterior restorations because of its great esthetic combined with high strength. The high translucency of LD enable the production of natural results even in cervical portion of the restoration where in the conventional metal-ceramic restorations, a dark shadow is likely to be visible. 14 Translucent zirconia and zirconia-reinforced lithium silicate (ZLS) are relatively new alternatives indicated for anterior restorations. Translucent zirconia was developed with increased yttria content to up to 5 mol% to overcome the aesthetic disadvantage of the material. 15 Sen and Isler 16 found that extra translucent zirconia produces comparable optical properties to that of LD. Cho et al 17 showed that compared to LD, 5Y-ZP had 80% translucency at 0.8 mm thickness and 89% at 1.5 mm thickness. Similarly, ZLS can produce satisfactory optical properties. 18 The material composed of lithium silicate as the main crystalline phase in a vitreous matrix reinforced with 10% dissolved zirconia (ZrO2). 18 The highly dispersed ZrO2 content is responsible for the generation of significantly more crystallization nuclei, which is supposed to present a higher ratio of the glass phase when compared with the conventional LD. 19 However, little is known about the marginal fitting of these materials compared to that of LD.

Several review papers on the marginal adaptation of fixed restorations showed that it is inconsistent, variant, and directly affected by the experimental protocol employed in investigating it. 20 21 These are caused by variations in study designs, measurement methods, and the adopted definitions of the marginal fitting. Therefore, comparing marginal discrepancy values across studies should be made with great caution. Instead, such comparisons could be made for different crown systems in one investigation under standardized method. To the authors' knowledge, there has been no previous research which compared the marginal adaptation of the five crown systems in one study. Hence, this study aimed to measure and compare marginal accuracy of five contemporary ceramic materials used for anterior restorations. Our null hypothesis indicates no statistically significant differences in their marginal gap.

## Materials and Methods

### Master Die and Crowns Fabrication

**Fig. 1:**
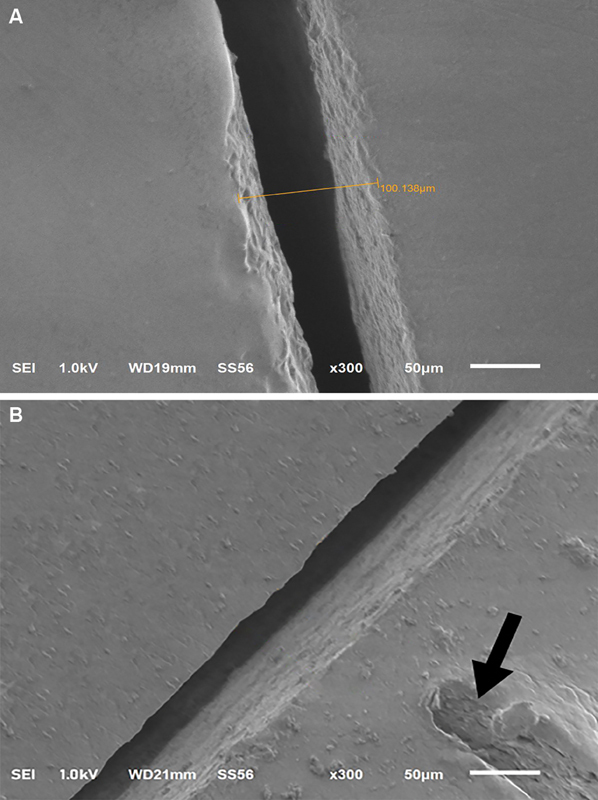
( **A** ) Vertical gap measured. ( **B** ) The arrow shows the indentation on the die identifying the point of measurement.

A master die (Nissin Dental Products INC, Kyoto, Japan) of maxillary central incisor was prepared following guidelines for all-ceramic crown preparation with an axial/incisal reduction of 1.5 mm and a chamfer of 1 mm width. The amount of tooth reduction was controlled using an index of the same tooth before preparation. The master die was marked with indentations placed external to the preparation finish line at mid-labial, mid-palatal, mid-mesial, and mid-distal points to standardize gap measurement points ( Fig. 1 ). Fifty impressions of the master die were made using silicone impression material (3M ESPE, St. Paul, Minnesota, United States) and molded with epoxy resin die material (Exakto-Form, Bredent, Germany) to produce 50 replicas of the master die.

The master die was sprayed with CEREC Optispray (Dentsply Sirona, Bensheim, Germany) and scanned by Cerec inEos X5 (Sirona Dental Systems, Bensheim, Germany). Scanning data were saved in STL (Standard Triangular Language) format to be used for the designing of the all-ceramic crowns (inLab CAM SW16, Dentsply Sirona) starting with the biogeneric design technique. The CAD system permits the adjustment of different parameters such as restorative material thickness and cement space. Therefore, the minimum thickness of the designed crown was set at 1 mm to correspond for the tooth preparation recommended for anterior ceramic crown, and the cement space was set at 50 µm. 22 Then, the designed crown was milled from five dental ceramics ( Table 1 ) using 5-axis milling machine (MC X5, Dentsply Sirona). Cercon xt and e.max ZirCAD were dry milled while the e.max CAD, Vita Suprinity and Celtra Duo were wet milled. The fit of crowns onto replica dies was controlled by a stereomicroscope (Wild M3C, Wild, Heerbrugg, Switzerland) with a magnification factor of 10. Cercon xt and e.max ZirCAD crowns were sintered following the manufacturer's guidelines (in fire HTC speed, Dentsply Sirona). The e.max CAD and Vita Suprinity crowns were crystallized in Programat EP 3010 (Ivoclar Vivadent, Schaan, Liechtenstein). The crowns were glazed and secured to epoxy resin dies with a droplet of temporary cement (RelyX Temp NE; 3M-ESPE) on the incisal edge. 23

**Table 1:** Details of the CAD/CAM materials evaluated in the study

Ceramic material	Composition	Manufacturer
e.max CAD	∼40 vol.% of ∼0.5 μm grain-size, lithium-metasilicate crystalline phase in a lithium disilicate glass	Ivoclar Vivadent, Schaan, Liechtenstein
e.max zirCAD	Yttrium oxide- (yttria-) stabilized zirconium oxide (Y-TZP).	Ivoclar Vivadent, Schaan, Liechtenstein
Celtra Duo	Lithium silicate glass with 10% dissolved zirconia. It also contain diphosphorus pentoxide to nucleate lithium metasilicate crystallization	Dentsply Sirona, Bensheim, Germany
Vita Suprinity	Lithium silicate glass with 10% dissolved zirconia. It also contain diphosphorus pentoxide to nucleate lithium metasilicate crystallization	Vita Zahnfabrik, Bad Säckingen, Germany
Cercon xt	Yttrium oxide- (yttria-) stabilized zirconium oxide (Y-TZP).	Dentsply Sirona, Bensheim, Germany

Abbreviation: CAD/CAM, computer-aided design/computer-aided manufacturing.

### Fit Measurement

**Fig. 2:**
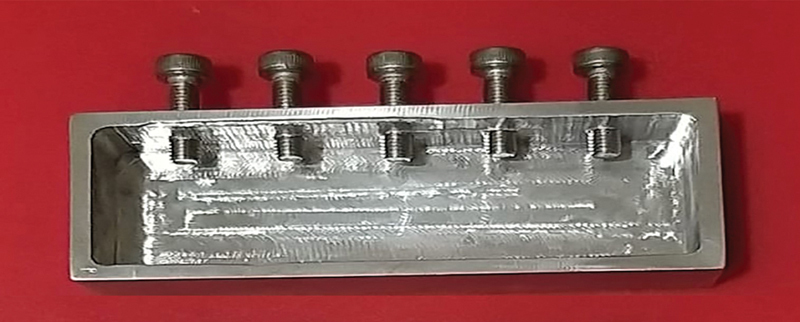
A custom-made jig to hold the crowns during marginal gap measurement.

Marginal accuracy of the restorations was evaluated by scanning electronic microscope (SEM) (JSM-6610LV, JEOL, United States) with a magnification of 300 × . Fit evaluation was made by measuring the vertical gap from the external crown margin to the opposite preparation line ( Fig. 1 ). Specimens were gold-coated by a Q15RS metallizer (Quorum Technologies, Sussex, United Kingdom) before SEM examination. The measurements were taken by fixing the specimens in a custom-made jig ( Fig. 2 ) placed perpendicular to the optical axis of the microscope. The marginal fit was measured for each crown at the predetermined four points.

### Statistical Analysis

Data was analyzed using SPSS software 23.0 (SPSS, Chicago, Illinois, United States). Shapiro–Wilk test confirmed the normal distribution of data. Descriptive statistics (mean and standard deviation) for the marginal gap and maximum gap values were performed. One-way analysis of variance was used to test differences between groups and Tukey test was used for multiple comparisons between group combinations. A level of significance at 95% was set for all statistics.

## Results

Means, standard deviations, and the mean maximum gap values of different materials are presented in Table 2 . There was a statistically significant difference between groups ( *p* < 0.5) in both mean marginal gaps and mean maximum gaps. The mean marginal gap of the e.max crowns (49.2 µm) was statistically significantly greater than those of all other groups ( *p* < 0.05). However, when the e.max CAD group was excluded, the differences between all other combinations were insignificant. Similar statistical results were obtained for the mean maximum gap comparisons where the highest value was for the e.max CAD (87.6 µm), which was statistically significantly greater than all other groups while all other combinations showed no significant differences.

**Table 2:** Mean marginal gap, standard deviation, and mean maximum gap of studied crowns

	e.max CAD	e.max ZirCAD	Celtra Duo	Vita Suprinity	Cercon xt
Mean marginal gap	49.3 ^a^	17.7 ^b^	13.2 ^b^	15.3 ^b^	10.2 ^b^
Standard deviation	33.4	12.6	17.0	14.0	9.0
Mean maximum gap	87.6 ^a^	32.9 ^b^	29.0 ^b^	32.1 ^b^	21.7 ^b^

Note: Different superscript in the same row indicates significant difference.

## Discussion

One spot with a large marginal discrepancy can determines the clinical risk of restoration. 24 Therefore, in addition to the mean marginal gap, the mean maximum gap values of the five tested material were reported and compared. There was statistically significant difference between the tested materials; hence, we rejected the null hypothesis.

Results showed that e.max ZirCAD and Cercon xt have a greater marginal precision compared to LD (e.max CAD) and were well below the maximum cement space of clinical acceptability identified by ADA. 9 Marginal gap values ranged between 0 and 75  μm were recorded for zirconia crowns 25 26 which is well within the acceptable range of 120 μm suggested by McLean and von Fraunhofer. 27 This might be linked to the precision of the CAD/CAM system in milling zirconia restorations, possibly because dental CAD/CAM systems were originally developed to process polycrystalline materials. 28 A recent systematic review found that the performance of a specific CAD/CAM system in terms of marginal adaptation is influenced by the type of restorative material. 11 Similarly, the two commercial examples of the ZLS (Vita Suprinity and Celtra Duo) presented superior results compared to e.max CAD. A previous study 29 agrees with our results as Vita Suprinity had significantly lower marginal discrepancy value (77 μm) than that of e.max CAD (130 μm). Though the mean marginal discrepancy values reported 29 were noticeably higher than those of the current study (Vita Suprinity, 15.5 and e.max CAD 49.2). On the contrary, Hasanzade et al 30 reported no significant difference between the two materials. Contradictions in gaps reported be different studies are expected and attributed to variances in study designs and protocols followed.

LD crowns showed the highest gap measured among the tested systems, which might influence its clinical survival compared to other systems. 4 A wide range of mean marginal discrepancy values of e.max CAD crowns were reported in previous studies with some of them being, according to McLean and von Fraunhofer, 27 clinically unacceptable: 87, 31 147.56, 32 63.73, 88.64, 33 125.46 to 135.59, 34 and 132.2 μm. 35 However, as mentioned earlier, these variations across studies are expected. The higher marginal discrepancy value of the e.max CAD crowns can be linked to ceramic shrinkage at the margin during crystallization firing. 36 Additionally, Fraga et al 37 found that surface roughness and defects after milling LD were more than those observed in zirconia.

**Fig. 3:**
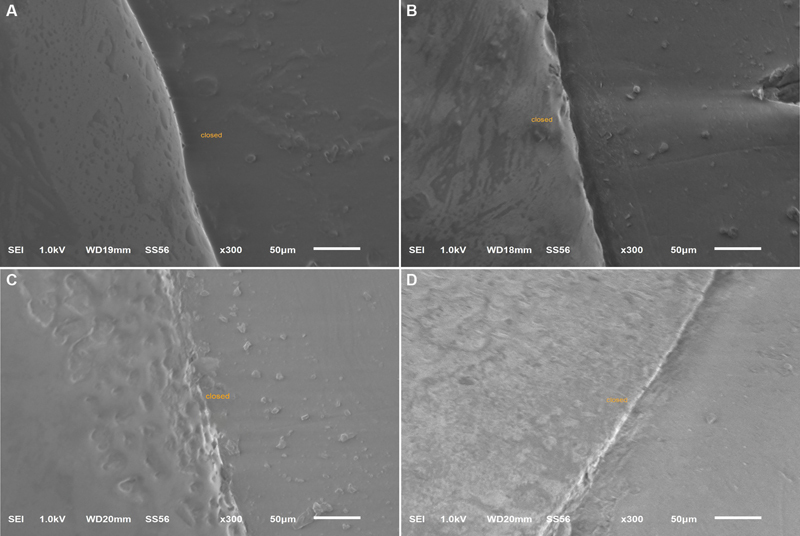
Perfectly closed margins. ( **A** ) Celtra Duo, ( **B** ) Vita Suprinity, ( **C** ) e.max ZirCAD, and ( **D** ) Cercon xt.

Theoretically, the precision of the designing and milling produced by contemporary CAD/CAM technology should produce a restoration with a marginal accuracy of zero discrepancy all around the margin, but this is known to be practically impossible. Though our SEM images showed a closed margin at several measurement points ( Fig. 3 ), which were more frequent in the zirconia systems. Boitelle et al 12 in a systematic review suggested that the available CAD/CAM technology delivers dental restorations with marginal discrepancy values of less than 80 µm, which is confirmed by the current study. In fact, this study found that the average maximum gap of all material except the e.max CAD were within the maximum range of the cement thickness identified by the ADA. 9

All crown systems in this study showed a clinically acceptable mean marginal gap and mean maximum gap of less than 120 μm. 27 The clinically acceptable marginal gap of fixed restorations has been a controversial subject in the literature. 9 27 38 39 A value of 120 μm which was established in 1971 27 is the most commonly cited value for clinical acceptability. Though such value should be revised as a marginal opening of 30 µm has been reported to encourage secondary caries formation. 4

In the current study, marginal fit was evaluated by measuring the vertical gap at the margin which might be considered a limitation because the absolute marginal discrepancy is the measurement that represents the total crown misfit at specific point, both vertically and horizontally. 40 However, the vertical and horizontal measurements have different clinical implications. 30

## Conclusion

Within the limitations of this study, it can be concluded that the marginal accuracy of LD crowns is clinically acceptable. Zirconia and ZLS materials can produce a greater level of marginal accuracy compared to LD.
